# Management of perioperative thyrotoxicosis – what to do when standard therapy is contraindicated or fails?

**DOI:** 10.3389/fendo.2024.1498014

**Published:** 2024-12-11

**Authors:** Kristy Tian, Trilene Liang, Jielin Yew, Chiaw-Ling Chng

**Affiliations:** ^1^ Department of Endocrinology, Singapore General Hospital, Singapore, Singapore; ^2^ Department of Endocrinology, Changi General Hospital, Singapore, Singapore

**Keywords:** perioperative, thyrotoxicosis, plasma exchange, Graves’ disease (GD), lithium, cholestyramine, beta blocker, glucocorticoids

## Abstract

**Background:**

Current guidelines recommend that hyperthyroid patients should be rendered euthyroid prior to surgical procedures. These guidelines rely heavily on the use of ATDs as the primary medication, and do not give recommendations for patients who have contraindications to ATDs, or for whom standalone ATD treatment is inadequate.

**Objectives:**

To evaluate the efficacy and safety of adjunctive pharmacological therapy and/or therapeutic plasma exchange (TPE) in the perioperative management of patients with thyrotoxicosis who were intolerant to ATD or for whom standalone ATD therapy was inadequate to achieve euthyroidism prior to surgery.

**Methods:**

A comprehensive search of MEDLINE, Google Scholar, Embase and CENTRAL up to 31 December 2023 retrieved 12,876 records. After screening titles, abstracts and full manuscripts, 16 reports were enrolled. The study quality was evaluated using the Newcastle-Ottawa Scale (NOS).

**Results:**

Patients were primarily female (78.5%), aged between 35 and 52 years. The predominant thyroid condition was Graves’ disease (89.7%). Majority underwent thyroidectomy (99.3%). Patients treated pre-operatively with 2nd line pharmacotherapy with/without therapeutic plasma exchange (TPE) underwent surgery safely with no reports of perioperative thyroid storm. Pre-operative treatment achieved mean percentage reduction of free thyroxine and free triiodothyronine levels of 52.6 ± 8.2% and 68.1 ± 9.3% respectively. One study reported a patient who suffered from myocardial infarction and tachyarrhythmia and subsequently demised.

**Conclusion:**

Preoperative management of hyperthyroidism with second line pharmacotherapy and/or TPE can be effectively and safely implemented in patients with intolerance to or ineffective treatment with ATDs. The treatment modalities were generally safe, though some complications were observed.

## Introduction

Preoperative thyrotoxicosis is a potentially life-threatening condition that requires medical intervention before surgery. The positive ionotropic and chronotropic effects of thyroid hormone on the heart culminate in an increase in cardiac output and arrhythmias. Elective surgeries should be postponed in patients with overt hyperthyroidism because of the risk of precipitating thyroid storm (TS) and potential cardiac complications. Although antithyroid drugs (ATDs) are the preferred initial treatment, clinical response may take weeks. There are potential limitations of ATDs in managing complex cases of thyrotoxicosis, such as in cases of ATD intolerance, where patients are unable to tolerate ATDs due to adverse effects such as agranulocytosis or hepatotoxicity, or ATD resistance (1–3), where there is failure to achieve or maintain euthyroidism despite adequate dosing and compliance with ATD therapy. Studies have reported the use of combination pharmacotherapy or therapeutic plasma exchange (TPE) to rapidly achieve clinical and biochemical euthyroidism in patients who require rapid optimization of thyrotoxicosis.

Second line pharmacotherapy in the management of thyrotoxicosis include the following: 1. Corticosteroids inhibit the peripheral conversion of thyroxine (T4) to the more active triiodothyronine (T3), helping to reduce the levels of T3, which is primarily responsible for the hypermetabolic symptoms of thyrotoxicosis (4). 2. Lithium inhibits the synthesis and release of thyroid hormones from the thyroid gland (5). 3. Cholestyramine binds thyroid hormone in the intestine and facilitates fecal excretion of thyroid hormone, thereby reducing enterohepatic recirculation and serum thyroid hormone levels (6). 4. Beta-blockers primarily mitigate the peripheral effects of excess thyroid hormone on the cardiovascular system. High dose Propranolol has been shown to inhibit peripheral conversion of T4 to T3, which further contributes to the amelioration of clinical symptoms and signs of thyrotoxicosis (7). Existing guidelines suggest initiation of beta-blockers when heart rate exceeds > 90 to aim for a normal pulse rate (8).

TPE, a class 2C recommendation for the treatment of TS by American society for apheresis (9), involves the extracorporeal removal of plasma containing excessive thyroid hormones, which are then replaced with donor plasma or albumin. The effectiveness of TPE in reducing thyroid hormone levels has been demonstrated in various studies, showing significant decreases in thyroid hormone levels post-treatment (10, 11).

### Rationale for this systematic review

Current guidelines (12, 13) advise that elective surgeries should always be postponed in patients with overt hyperthyroidism. The published guidelines by the American Thyroid Association also recommends that patients undergoing thyroidectomy for Graves disease should be rendered euthyroid prior to the procedure (8). Patients who require emergency surgeries should be premedicated and monitored closely for complications perioperatively. However, these guidelines rely heavily on the use of ATDs as the primary medication, and do not give recommendations for patients who have contraindications to ATDs, or for whom standalone ATD treatment is inadequate. There are no systematic reviews or Cochrane reviews on this topic.

## Materials and methods

### Objectives

The aim of this systematic review is to evaluate the efficacy and safety of adjunctive pharmacological therapy and/or therapeutic plasma exchange (TPE) in the perioperative management of patients with thyrotoxicosis who were intolerant to ATD or for whom standalone ATD therapy was inadequate to achieve euthyroidism prior to surgery. The latter group comprised patients who either remained hyperthyroid despite maximal ATD treatment or required rapid optimization of thyrotoxicosis necessitating the use of adjunctive treatment alongside ATD therapy.

### Methods

#### Data sources, search strategy and selection criteria

This systematic review utilized the Preferred Reporting Items for Systematic Reviews and Meta-Analyses (PRISMA) guideline for a systematic literature search.

We included studies reporting on hyperthyroid patients going for surgery who were intolerant to ATD therapy or for whom standalone ATD therapy was inadequate. We divided the studies describing patients who received an intervention into four groups:

- Patients with intolerance to ATDs – treated with 2^nd^ line pharmacotherapy.
- Patients with intolerance to ATDs – treated with TPE with/without 2^nd^ line pharmacotherapy.
- Patients for whom standalone ATD therapy was inadequate – treated additionally with 2^nd^ line pharmacotherapy.
- Patients for whom standalone ATD therapy was inadequate – treated additionally with TPE with/without 2^nd^ line pharmacotherapy.

Studies unrelated to human beings or on people <18 years old were excluded. The primary outcomes are thyroid storm, cardiovascular morbidity and mortality. The secondary outcomes are changes in thyroid hormone (free thyroxine (fT4) and free triiodothyronine (fT3)) levels.

We searched the Cochrane Register of Controlled Trials (CENTRAL), Embase, PubMed/MEDLINE, and Google Scholar databases for relevant studies. The following terms were connected using Boolean operators “and”, “or”, “and/or”, “hyperthyroidism”, “thyrotoxicosis”, “Graves disease”, “perioperative care”, “anaesthesia”, “operation”, “beta-blockers”, “glucocorticoids”, “lithium”, “cholestyramine”, “iodine”, “plasma exchange”. The terms were searched as “Mesh terms” and as “all fields” terms.

Two reviewers independently screened the titles and abstracts of articles retrieved from the databases. Articles were labelled as “include”, “uncertain” or “exclude”. Articles were included for full-text screening if both reviewers labelled the articles as “include”. Articles were excluded from full-text screening if both reviewers labelled the article as “exclude”. Disagreements were resolved by consensus.

Two reviewers then independently screened the full-texts of shortlisted articles for eligibility for inclusion in the systematic review. Articles were labelled as “include”, “uncertain” or “exclude”. Articles were included for data extraction if both reviewers labelled the articles as “include”. Articles were excluded from data extraction if both reviewers labelled the article as “exclude”. Disagreements were resolved by consensus.

#### Data collection and quality assessment

Four reviewers independently reviewed the full text of the studies and extracted data on the authors, year of publication, location of study, study design, setting, number of participants, number of dropouts, age of participants, details on pharmacological intervention, and outcomes. The outcomes included thyroid storm, cardiovascular morbidity, mortality and change in thyroid hormone levels. The study quality was evaluated using the Newcastle-Ottawa Scale (NOS), which is based on selection (4 items), comparability (1 item) and outcome (3 items). It provides a rating system ranging from 0 to 9, with scores ≥ 7-9, 4-6 and <4 considered low, intermediate, and high risk of bias respectively (14).

## Results

**Figure 1:**
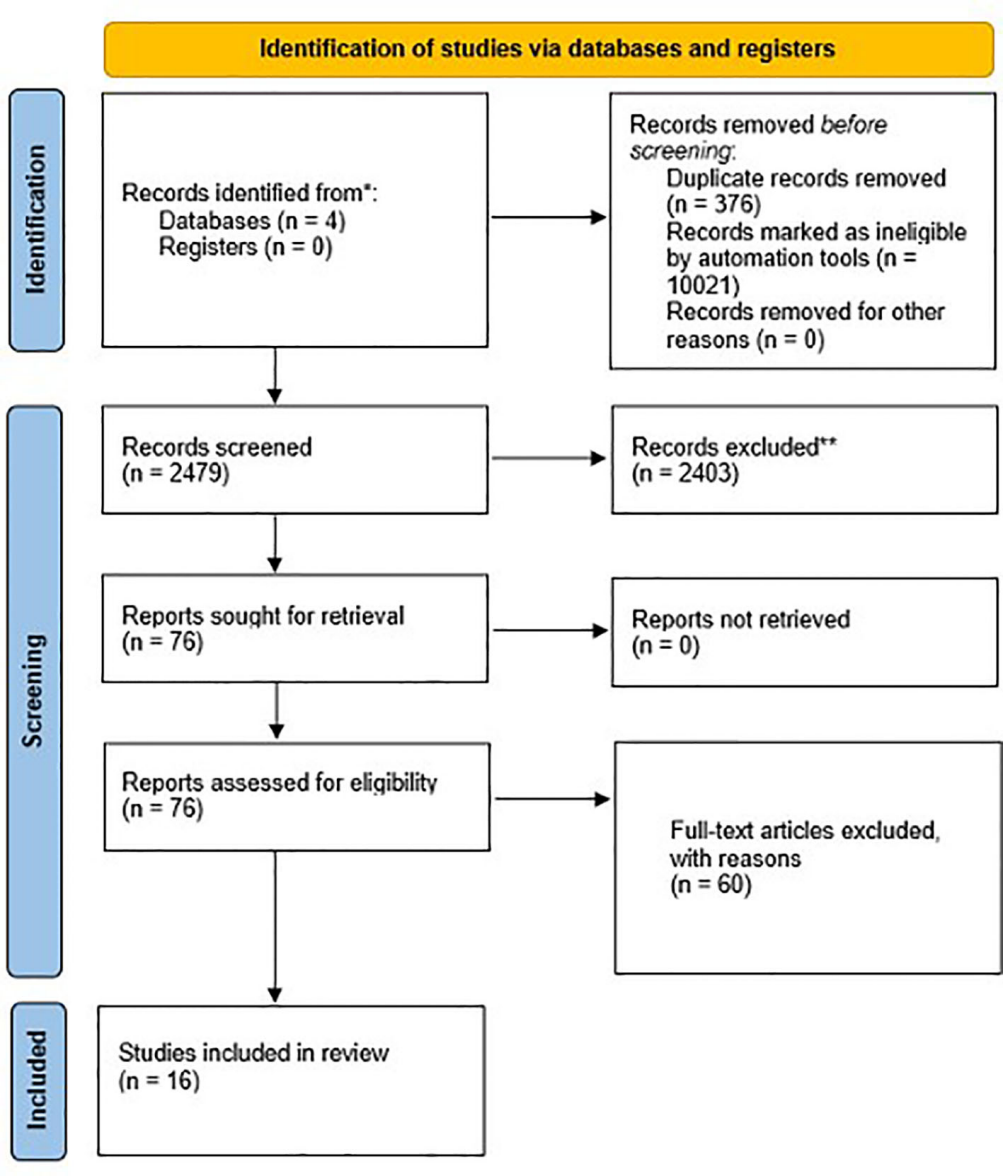
Study flow diagram.

For the study, 12,876 records were retrieved from MEDLINE (3335), Google Scholar (1010), Embase (8327) and CENTRAL (Cochrane Central Register of Controlled Trials) (204). The study flow diagram is shown in **Figure 1**. The complete list of articles obtained through the systematic search was screened to remove duplicates and exclude ineligible articles. Seventy-six studies were selected from title and abstract screening and retrieved for full-text screening. Sixty studies were deemed ineligible and 16 studies were included in the systematic review. Study quality was assessed using NOS; two studies obtained 7 points, twelve obtained 6 points, and the remaining 2 obtained 5 points (**Table 1**).

**Table 1:** Newcastle-Ottawa scale of studies included in the systematic review.

Study	Selection				Comparability	Outcome			Total
	Representativeness of the exposed cohort	Selection of non-exposed cohort	Ascertainment of exposure	Demonstration that outcome was not present at start of study	Comparability of cohorts	Assessment of outcome	Follow up duration	Adequacy of follow up	
Kirkizlar et al	*		*	*		*		*	5
Durmus et al	*		*	*		*	*	*	6
Kartal et al	*	*	*	*		*	*	*	7
Builes et al	*		*	*		*	*	*	6
Yabanoglu et al	*		*	*		*	*	*	6
Saie et al	*		*	*		*	*	*	6
Ozdemir et al	*		*	*		*	*	*	6
Ali et al	*	*	*	*		*	*	*	7
Simsir et al	*		*	*		*	*	*	6
Calissendorff et al	*		*	*		*		*	5
Fischli et al	*		*	*		*	*	*	6
Keklik et al	*	*	*	*		*	*	*	6
Ezer et al	*		*	*		*	*	*	6
Panzer et al	*		*	*		*	*	*	6
Apaydin et al	*		*	*		*	*	*	6
Akin et al	*		*	*		*	*	*	6

(*) indicates a point that can be awarded for an answer to a question

### Findings

The average age of patients was between 35 and 52 years. Majority of patients were female (78.5%). The most common underlying thyroid condition was Graves disease (89.7%), followed by toxic nodular goiter (TNG) (7.3%). The most common operation performed was thyroidectomy (99.3%). Four patients underwent non thyroid surgery – 2 patients underwent fixation of femoral fractures, 1 underwent cardiac surgery and 1 underwent abdominal surgery. The baseline demographics of patients in the studies are shown in **Table 2**.

**Table 2:** Summary of included studies and indications of 2nd line treatment.

Year	Title	No. of pts	Female gender (%)	Underlying thyroid condition			Indication for 2^nd^ line treatment		Type of operation		2^nd^ line agents used
				Graves’ Disease	TNG	Others*	ATD intolerance	Insufficient response to ATD	Thyroidectomy	Others	
2022	Therapeutic plasma exchange in hyperthyroidism prior to surgery	11	10 (90.9%)	7	4	0	7	4	10	1	Beta blockers, TPE
2022	Efficacy of preoperative therapeutic plasma exchange in patients with hyperthyroidism and factors affecting the number of sessions	21	12 (57.1%)	20	1	0	19	2	20	1	Beta blockers, steroids, cholestyramine, lithium, iodine, TPE
2022	Comparison of urgent and elective thyroidectomy complications in Graves’ disease	113	92 (81.4%)	113	0	0	12	0	113	0	Steroids, iodine
2021	Therapeutic plasmapheresis for the treatment of thyrotoxicosis: A retrospective multi-center study	19	11 (57.9%)	16	3	0	8	0	10	1	Beta blockers, steroids, cholestyramine, iodine
2021	Preoperative Therapeutic Plasma Exchange and Surgical Treatment in Thyrotoxicosis Patients: A Single-Centre Retrospective Cohort Study	27	15 (55.6%)	16	7	4	6	15	21	0	TPE
2020	Therapeutic Plasma Exchange in Refractory Hyperthyroidism	22	12 (54.5%)	13	1	8	13	9	16	0	Steroids, cholestyramine, TPE
2020	The role of therapeutic plasmapheresis in patients with hyperthyroidism	18	11 (61.1%)	9	8	1	15	0	13	2	Beta blockers, steroids, TPE
2019	Outcomes After Urgent Thyroidectomy Following Rapid Control of Thyrotoxicosis in Graves’ Disease are Similar to Those After Elective Surgery in Well-Controlled Disease	266	228 (85.7%)	266	0	0	5	14	266	0	Beta blockers, steroids, cholestyramine
2018	Therapeutic plasmapheresis in thyrotoxic patients	46	32 (69.6%)	40	2	4	21	19	40	0	TPE
2017	Rescue pre-operative treatment with Lugol’s solution in uncontrolled Graves’ disease	27	25 (92.6%)	27	0	0	22	5	26	0	Beta blockers, iodine
2016	Rapid preoperative blockage of thyroid hormone production/secretion in patients with Graves’ disease	10	7 (70.0%)	10	0	0	8	2	10	0	Beta blockers, steroids, iodine
2013	The results of therapeutic plasma exchange in patients with severe hyperthyroidism: a retrospective multicenter study	22	16 (72.7%)	9	13	0	22	0	5	0	Beta blockers, steroids, TPE
2009	Preoperative therapeutic plasma exchange in patients with thyrotoxicosis	11	8 (72.7%)	7	3	1	2	7	10	1	Beta blockers, TPE
2004	Rapid preoperative preparation for severe hyperthyroid Graves’ disease	17	16 (94.1%)	12	4	1	3	0	17	0	Beta blockers, steroids, iodine
2020	Preoperative plasmapheresis experience in Graves’ disease patients with anti-thyroid drug-induced hepatotoxicity	27	15 (55.6%)	5	0	0	6	15	5	0	Beta blockers, steroids, cholestyramine, TPE
2007	The use of lithium carbonate in the preparation for definitive therapy in hyperthyroid patients	6	5 (83.3%)	5	1	0	3	1	3	0	Beta blockers, steroids, lithium

*17 cases of amiodarone induced thyrotoxicosis, 1 case of non-autoimmune TSH receptor activation, 1 case of iodine induced thyrotoxicosis.

#### Patients with ATD intolerance – treated with 2^nd^ line pharmacological therapy

Five studies reported a total of 47 patients with ATD intolerance requiring 2^nd^ line pharmacological therapy preoperatively. The complications experienced with ATD treatment include hepatotoxicity, agranulocytosis, vasculitis and allergic reactions. There were also reports of patients unable to tolerate ATD treatment due to arthritis, thrombocytopenia and drug-drug reactions. *Kartal* et al. reported 9 patients who received 2^nd^ line pharmacological therapy (6 received Lugol’s iodine, 1 received Lugol’s iodine and steroids, 2 received steroids) for mild hyperthyroidism due to ATD intolerance (15). *Ali* et al. reported 4 patients who received cholestyramine and beta blockers, and 1 patient who received cholestyramine, beta blockers and steroids due to ATD intolerance (16). Both authors compared the group of patients with ATD intolerance requiring 2^nd^ line pharmacological therapy for rapid preoperative optimization against patients with well controlled hyperthyroidism who underwent elective surgery. All patients underwent surgery safely with no events of thyroid storm reported. There was no significant difference in clinical outcomes between the rapid optimization and elective surgery groups. *Calissendorff* et al. reported twenty-two patients who received Lugol’s iodine and beta blocker with improvements in thyroid hormone levels and successfully underwent total thyroidectomy (17). Reported adverse effects of iodine treatment were rash (n=2), rash and vomiting (n=1) and swelling of fingers (n=1) - these were managed with dose reduction in two and stopping Lugol’s iodine prematurely in 2. *Fischli* et al. reported eight patients who received a combination of Lugol’s iodine, beta blockers and dexamethasone and successfully underwent thyroidectomy (18). In this study, dexamethasone was given twice daily at 2mg/day for 10-14 days (including the postoperative period) as per a previously reported protocol (19). Three patients showed prolonged secondary adrenal insufficiency with normalization of adrenal function after 3 to 6 months. Only one study by *Akin* et al. reported the use of lithium in 3 patients with underlying Graves’ disease and ATD intolerance (20) – patients achieved euthyroidism in 2-3 weeks and successfully underwent thyroidectomy with successful outcome.

#### Patients with ATD intolerance – treated with TPE with/without 2^nd^ line pharmacological therapy

Eleven studies reported 131 patients who received preoperative treatment with TPE due to ATD intolerance. The complications experienced with ATD treatment include hepatotoxicity, agranulocytosis, vasculitis and severe cutaneous reactions. Most patients started on TPE were concurrently treated with 2^nd^ line pharmacotherapy including beta blockers, corticosteroids, cholestyramine, lithium and iodine. Ten of the studies reported an average of 3-6 sessions of TPE required preoperatively. *Durmus* et al. reported a higher number of TPE sessions required (10.10 ± 7.17 sessions) (21). Patients in this study also had higher pre-treatment fT4 (56.1pmol/L) and fT3 (23.7pmol/L) levels compared to the other studies (mean fT4 48.0pmol/L, mean fT3 20.6pmol/L). It was also shown in the study that pretreatment fT3 level is a factor that predicts for higher number of TPE sessions. In addition, all patients in this study were intolerant of ATDs, and the author proposed that the lack of the ATD suppressive effect on autoimmunity may have contributed to the more resistant elevation of thyroid hormone levels, leading to higher number of TPE sessions required. *Keklik* et al. reported 22 patients (9 with underlying Graves’ disease, 13 with underlying TNG) with ATD intolerance who underwent a median of 4 TPE sessions. One patient (60-year-old female) with underlying TNG suffered a myocardial infarction and tachyarrhythmia, presumably as a complication of thyrotoxicosis, and subsequently passed away before undergoing thyroidectomy. Two studies reported the use of fresh frozen plasma (FFP) only, 1 study reported the use of albumin only, 6 studies reported the use of either FFP or albumin and 2 studies did not specify the exchange fluid used. Complications from treatment were largely mild allergic reactions that were managed medically. One patient developed anaphylaxis to FFP requiring a change of exchange fluid to albumin. Three patients developed catheter related complications of infection and deep vein thrombosis. *Kirkzlar* et al. reported the successful use of TPE in the second trimester of pregnancy for a patient who had PTU induced angioneurotic edema, with successful thyroidectomy performed in the 20^th^ week of pregnancy (22).

#### Patients for whom standalone ATD therapy was inadequate – additional treatment with 2^nd^ line pharmacological therapy

Four studies reported a total of 22 patients receiving additional 2^nd^ line pharmacological therapy due to inadequate ATD response. All patients had underlying Graves’ disease. Patients were treated with a combination of beta-blockers, cholestyramine, Lugol’s iodine, lithium and steroids. *Ali* et al. described 14 patients who had inadequate response to ATD therapy and required additional 2^nd^ line pharmacological therapy for rapid optimization of Graves’ disease preoperatively (16). The clinical outcomes of these patients were compared to 247 patients who underwent elective surgery for well-controlled Graves’ disease. The median free thyroid hormone levels were higher in the rapidly optimized group as compared to the well-controlled group (fT4 40pmol/L vs. 15pmol/L (reference range 12-22pmol/L), fT3 15pmol/L vs. 5pmol/L (reference range 3.1-6.8pmol/L)). Majority of patients in the rapidly optimized group demonstrated reduction in free thyroid hormone levels after combination pharmacological therapy was initiated, but only 42% and 53% of patients had normal fT4 and fT3 levels, respectively, prior to surgery. There was no report of thyroid storm. There were also no significant differences in the clinical outcomes between both groups. The authors proposed a protocol for the rapid optimization of thyroid function preoperatively in poorly controlled Graves disease unable to achieve euthyroidism with ATDs, with suggestion to add Lugol’s iodine, propranolol, cholestyramine and dexamethasone in a stepwise fashion, targeting fT4 <30pmol/L (reference range 12-22pmol/L) and fT3 <10pmol/L (reference range 3.1-6.8pmol/L) prior to operation. *Calissendorff* et al. reported 5 patients who received additional therapy with Lugol’s iodine and propranolol 7-10 days preoperatively due to poor adherence to ATDs (17). All patients were admitted for the last part of treatment (6 days (0–12)) and successfully underwent total thyroidectomy uneventfully despite thyroid hormones not completely normalizing (fT4 20 (range: 8-52), fT3 (6.5 (range: 4.3-8.3)), reference ranges for fT4 8-14pmol/L, fT3 3.5-5.4pmol/L). *Fischli* et al. described the addition of Lugol’s iodine, dexamethasone and a beta-blocker (propranolol, metoprolol or bisoprolol) in 2 patients with Graves’ disease who were non-compliant to carbimazole (18). Surgery was performed successfully 10 days after starting treatment. *Akin* et al. reported a patient with Graves’ disease who remained hyperthyroid despite propylthiouracil dose of 800mg/day, and thus was additionally commenced on dexamethasone (1mg/day) and lithium carbonate. She became euthyroid after 2 weeks of combination therapy and underwent thyroidectomy successfully (20). Of note, a heart rate target of less than 80 beats per minute was achieved in 2 of 4 of the above studies.

#### Patients for whom standalone ATD therapy was inadequate – additional treatment with TPE with/without 2^nd^ line pharmacological therapy

Seven studies reported a total of 70 patients who received additional treatment with TPE and 2^nd^ line pharmacological therapy due to inadequate ATD response. Patients were concurrently treated with a combination of beta blockers, corticosteroids, cholestyramine and iodine. *Saie* et al. reported the indications of arrhythmias, thyrocardiac disease and thyroid eye disease requiring surgery in this group of patients for whom ATDs were inadequate at controlling hyperthyroidism (23). Again, majority of patients underwent thyroidectomy for underlying Graves’ disease or toxic nodular goiter. *Baser* et al. reported 2 patients who were unable to achieve euthyroidism with pharmacological therapy (methimazole, steroids and beta blockers), and underwent concurrent TPE prior to non-thyroid emergency surgery (cardiovascular and orthopaedic surgeries) (11). There were no reported outcomes of thyroid storm, cardiovascular morbidity or mortality. *Yabanoglu* et al. reported a higher rate of surgical complications (hypocalcaemia, haemorrhage and surgical site infections) and attributed this to the effects of TPE (24).

## Discussion

The current recommended goal of therapy in patients with hyperthyroidism is to render patients as close to clinical and biochemical euthyroidism prior to surgery, and current guidelines propose the use of ATDs as first line therapy in doing so. While most patients tolerate and respond well to ATDs, the development of serious complications precludes its use in certain scenarios. Severe hepatotoxicity and neutropenia are widely regarded as contraindications to ATDs in view of the potential devastating consequences. Other cited reasons for ATD intolerance include adverse events of vasculitis, severe cutaneous reactions, allergic reactions, arthralgia and drug-drug interaction with warfarin. Concerns of safe ATD usage in patients with pre-existing hepatic dysfunction have also prompted the use of 2^nd^ line pharmacotherapy in some studies (20).

ATDs may not be sufficient for patients who require rapid control of hyperthyroidism prior to surgery, and this group would benefit from combination pharmacotherapy or TPE to adequately prepare them for surgery. In clinical practice, inadequate control of hyperthyroidism is often the result of suboptimal adherence to ATDs. True ATD resistance as the cause of inadequate control of hyperthyroidism is rare and largely limited to case reports (25). There is no clear definition of ATD resistance, but reports have suggested that this diagnosis can be made in patients whose thyroid hormone levels remain elevated despite compliance to high doses of ATD (26) or when ATD doses exceeding that of standard dose ranges (up to 30mg/day for MMI and 300mg/day for PTU) are required to achieve euthyroidism (27). Risk factors for ATD resistance include large goitres, iodine excretion of ≥ 100 µg/g creatinine, high pretreatment thyroid hormone levels and presence of elevated TSH receptor antibody levels (28). Whilst exact mechanisms behind resistance to ATDs are poorly understood, possible aetiologies include malabsorption, rapid metabolism of the drug, anti-drug antibodies and defects in intrathyroidal accumulation or action (1). Elevated levels of MDR-1 multi-drug resistance gene may also be associated with disease activity and treatment resistance in patients with Graves disease (29). In patients with ATD resistance, doses of up to 150mg of MMI have been prescribed in combination with lithium, steroids and inorganic iodine prior to definitive therapy (26).

It is precisely the above group of patients who require definitive treatment of their hyperthyroidism due to intolerance to or inadequate treatment with ATDs. Total thyroidectomy would be the procedure of choice in certain patient populations (30). It is also not uncommon that patients with thyrotoxicosis require surgery for an unrelated condition. There is currently no risk stratification tool to predict the risk of complications such as thyroid storm, cardiovascular morbidity and mortality in hyperthyroid patients undergoing surgery. Patients who had a diagnosis of hyperthyroidism, presence of antithyroglobulin antibody and high thyroglobulin levels (>150 ng/mL) were identified to be at higher risk of surgical complications such as damage to the recurrent laryngeal nerve, damage to the parathyroid glands, and postoperative hematoma (31). However, thyroid storm, cardiovascular morbidity and mortality were not analysed in this paper. In a study done in a cohort of older orthopaedic patients, postoperative complications (e.g. cardiovascular events, cardiac failure, infections, stroke, and neurological complications) appeared to be higher in patients who were biochemically hyperthyroid, although incidence of thyroid storm was not reported (32).

Notably, there are no specific pre-operative biochemical cut-offs (8, 33) or clinical parameters that can reliably predict the prevention of thyroid storm. If urgent surgery is indicated, achieving a normal thyroid function before surgery may not be feasible. TSH values may remain suppressed even in patients with normalized T4 and T3 levels. Nonetheless, pre-operative treatment can be undertaken to minimise the risks of perioperative complications from thyrotoxicosis as evident from the studies included in this systematic review. Following pre-operative treatment, there was a mean percentage reduction of fT4 and fT3 by 52.6 ± 8.2% and 68.1 ± 9.3% respectively in the included studies (**Table 3**) and there were no events of perioperative thyroid storm reported despite a lack of normalization of thyroid hormone levels in some patients prior to thyroidectomy. Studies by *Ali* et al. and *Builes-Montaño* et al. did not report any events of perioperative thyroid storm despite just above 40% and 50% achieving normal fT4 and fT3 respectively prior to thyroidectomy. *Keklik* et al. reported a mortality from myocardial infarction complicated by tachyarrhythmia. This patient was older (60 years vs. median age of 47 years in the study) and had higher pre TPE thyroid hormone levels (pre-TPE fT4 75.2pmol/L vs. 44.2pmol/L and pre-TPE fT3 22.3pmol/L vs. 20.4pmol/L); though there were no details provided on patient’s baseline cardiac status and events surrounding the patient’s demise. Whilst *Calissendorff* et al. suggested a heart rate target of < 80 prior to thyroidectomy, optimal heart rate targets are uncertain. Elevated preoperative resting heart rate (> 87 beats/min) appeared to significantly correlate to postoperative myocardial impairment and mortality in patients who underwent non-cardiac surgery, although this was not specifically studied in a thyrotoxic population going for thyroidectomy (34). Existing guidelines suggest initiation of beta-blockers when heart rate exceeds > 90 to aim for a normal pulse rate (8) and higher rates of intraoperative tachycardia may be seen if normal pulse rates were not achieved preoperatively (35).

**Table 3:** Summary of mean changes (%) of fT4 and fT3 levels.

Author	Title	Mean change in fT4 levels (pmol/L)			Mean change in fT3 levels (pmol/L)		
		Pre	Post	% change	Pre	Post	% change
Kirkizlar et al	Therapeutic plasma exchange in hyperthyroidism prior to surgery	45.6	23	49.6	17.6	7.2	59.1
Durmus et al	Efficacy of preoperative therapeutic plasma exchange in patients with hyperthyroidism and factors affecting the number of sessions	56.1	18.2	67.6	23.7	5.7	75.9
Kartal et al	Comparison of urgent and elective thyroidectomy complications in Graves’ disease	41.2	21.9	46.8	20.4	7.2	64.7
Builes et al	Therapeutic plasmapheresis for the treatment of thyrotoxicosis: A retrospective multi-center study	52.1	23.3	55.3	NA	NA	NA
Yabanoglu et al	Preoperative Therapeutic Plasma Exchange and Surgical Treatment in Thyrotoxicosis Patients: A Single-Centre Retrospective Cohort Study	34.0	21.0	38.2	12.0	6.0	50.0
Saie et al	Therapeutic Plasma Exchange in Refractory Hyperthyroidism	68.0	NA	NA	21.0	NA	NA
Ozdemir et al	The role of therapeutic plasmapheresis in patients with hyperthyroidism	55.9	24.6	56.0	21.6	5.7	73.9
Ali et al	Outcomes After Urgent Thyroidectomy Following Rapid Control of Thyrotoxicosis in Graves’ Disease are Similar to Those After Elective Surgery in Well-Controlled Disease	40.0	21.9	45.3	15.0	6.4	57.3
Simsir et al	Therapeutic plasmapheresis in thyrotoxic patients	37.3	20.6	44.8	15.2	6.1	59.6
Calissendorff et al	Rescue pre-operative treatment with Lugol’s solution in uncontrolled Graves’ disease	53.0	20.0	62.3	20.0	6.5	67.5
Fischli et al	Rapid preoperative blockage of thyroid hormone production/secretion in patients with Graves’ disease	68.9	26.7	61.2	30.0	6.1	79.7
Keklik et al	The results of therapeutic plasma exchange in patients with severe hyperthyroidism: a retrospective multicenter study	44.2	20.4	53.8	26.5	8.2	69.1
Ezer et al	Preoperative therapeutic plasma exchange in patients with thyrotoxicosis	44.5	25.0	43.8	27.8	6.6	76.3
Panzer et al	Rapid preoperative preparation for severe hyperthyroid Graves’ disease	NA	NA	NA	NA	NA	NA
Apaydin et al	Preoperative plasmapheresis experience in Graves’ disease patients with anti-thyroid drug-induced hepatotoxicity	46.0	20.3	55.9	22.4	4.9	78.0
Akin et al	The use of lithium carbonate in the preparation for definitive therapy in hyperthyroid patients	53.2	23.2	56.4	16.1	4.3	73.6

NA, not reported.

While guidelines suggest for normalization of thyroid function prior to surgery, this may not be viable for patients with time-critical surgeries. This could also lead to delays in patients obtaining surgical treatment. In our review, patients with hyperthyroidism who were treated with second-line pharmacotherapy before surgery were able to undergo the procedure safely, even though their thyroid hormone levels did not normalize. Second line pharmacotherapy used as an alternative to or combination with ATD was safe in the studies included in this review. Side effects of rash, vomiting and finger swelling experienced with iodine were reversible with either dose reduction or stopping the offending medication. Three of 10 patients who received dexamethasone at 2mg/day for 10-14 days developed secondary adrenal insufficiency; all demonstrated normalization of adrenal function after 3-6 months. TPE was also an effective treatment option for pre-operative hyperthyroidism, achieving a mean percentage reduction in fT4 and fT3 levels of 51.7 ± 8.7% and 67.7 ± 10.3% respectively in the papers included in our review. Adverse effects of TPE in the studies were mostly mild: pruritus, rash, prolonged prothrombin time with no clinical bleeding manifestations and transient hypotension were reported. One patient suffered anaphylaxis to FFP, 2 had catheter-related infections, and 1 developed deep vein thrombosis. Based on the latest edition of American Society for Apheresis (ASFA) guidelines (9), TPE is currently indicated for treatment of thyroid storm in patients who respond poorly or have intolerance to first-line therapies (Category II, Grade 2C Evidence). We may consider broadening the criteria for using TPE to include patients who are not in thyroid storm, but require optimization of preoperative hyperthyroidism.

The NOS is a tool designed to assess the reliability and validity of findings reported in non-randomized studies included in systematic reviews. It gauges various aspects of study quality, including the selection of participants, the comparability of groups, and the assessment of outcomes. The studies included in our review generally demonstrated strong performance in the selection and outcome assessment components of the NOS. The cohorts were somewhat representative of the population of patients requiring perioperative management of thyrotoxicosis, though some studies were on patients with more severe disease requiring second-line pharmacotherapy and/or TPE. Exposure assessment was adequately conducted and outcomes were monitored across the perioperative period with complete follow up for all subjects. However, only three studies included a comparative non-exposed cohort, and none of these studies controlled for confounding factors such as thyroid hormone levels or comorbidities that could affect operative outcomes.

There are several limitations of this systematic review. Firstly, studies included in this review were either case-series or retrospective cohort studies which were largely descriptive in nature. Patient numbers are too few to extrapolate outcomes to a larger population and hence may not be generalisable. Secondly, due to heterogeneity of the studies and lack of head-to-head comparison studies, we were unable to ascertain which 2nd-line treatment option or combination was superior. Individual patient results were often not available and there were multiple variables that could have confounded outcomes. Thirdly, as studies included in this review were from various centres across the world with different reference ranges for thyroid hormone levels, we could only report the percentage change in thyroid hormone levels rather than absolute values. Thus, we were unable to identify any specific biochemical cut-offs. Further prospective studies are required to help risk-stratify patients who are at high risk of developing complications post operatively and evaluate safe threshold levels of thyroid hormone levels prior to thyroidectomy. Upcoming potential treatment options being studied including antigen specific immunotherapy e.g. ATX-GD-59 (36), monoclonal antibodies e.g. Iscalimab and TSH receptor-Blocking Antibodies e.g. IMVT-1402 could potentially be investigated as perioperative adjunctive treatment in the future (37, 38).

## Conclusion

There is a paucity of data regarding optimal therapy when conventional treatment with ATDs is not tolerated, is ineffective or when rapid optimisation is required for urgent surgery. Patients with hyperthyroidism should be treated to reduce the risk of thyroid storm and cardiac complications, although the most optimal 2^nd^ line regime is still uncertain and there is no available guidance in terms of biochemical cut-offs nor clinical parameters that are currently suggested in guidelines. Second line pharmacotherapy and/or TPE in this review were shown to be effective at reducing thyroid hormone levels to allow patients to safely proceed for surgery. More studies are needed to ascertain the optimal regime of 2^nd^ line therapy, develop risk assessment tools for patients at higher risk of post-operative thyroid storm and determine biochemical and clinical parameter cut-offs.

## Data Availability

The original contributions presented in the study are included in the article/supplementary material. Further inquiries can be directed to the corresponding author.
